# Efficacy of *Berberis vulgaris* and *Berberis* *integerrima* on glycemic indices and weight profile in type 2 diabetic patients: A systematic review and meta-analysis of randomized controlled trials

**DOI:** 10.1016/j.jaim.2024.101039

**Published:** 2024-10-15

**Authors:** Hassan ul Hussain, Eman Ali, Areesha Tanveer, Syed Muhammad Ismail, Humam Furqan, Muhammad Nadeem Ahsan, Sadia Iqbal, Muhammad Sohaib Asghar

**Affiliations:** a Dow University of Health Sciences, Karachi, 74200, Pakistan; b Mayo Clinic, Rochester, MN, USA

**Keywords:** Berberis, Type 2 diabetes mellitus, Blood glucose, Weight, Post-prandial, Serum insulin, Glycemic index

## Abstract

Type 2 diabetes mellitus (T2DM) is a multifactorial lifelong condition. *Berberis vulgaris* (BV) and *Berberis integerrima* (BI) regulate glucose levels with minimal adverse effects. In this systematic review and meta-analysis, we evaluate the effect of BV and BI on glycemic indices, weight, and body mass index (BMI) against placebo.

Four electronic databases were searched till September 24, 2023. Inclusion criteria for studies were: (a) T2DM patients; (b) berberis (vulgaris/integerrima) therapy as intervention; (c) control group of placebo or metformin; (d) outcomes including fasting blood glucose (FBG) levels, glycated hemoglobin (HbA1c%), Homeostatic Model Assessment for Insulin Resistance (HOMA-IR), fasting serum insulin (FSI), 2-h postprandial glucose (2hPPG), fructosamine, weight, and BMI; (e) randomized controlled trials (RCTs). Data was pooled using a random-effects analysis model on Review Manager. The inverse variance statistical method was applied keeping weighted mean difference (WMD) as the effect measure. The Cochrane risk of bias tool evaluated the risk of bias. A p-value of less than 0.05 was considered significant.

Nine RCTs comprising 547 patients were included. Significant reduction was noted with berberis therapy in FBG (WMD: −14.52; 95% CI = −22.97, −6.07; P = 0.0008); HbA1c % (WMD: −0.30; 95% CI = −0.53, −0.07; P = 0.01); HOMA-IR (WMD: −0.97; 95% CI = −1.56, 0.37; P = 0.001). No significant differences were noted between the two groups in 2hPPG (WMD: 6.52; 95% CI = −21.57, 34.61; P = 0.65); FSI (WMD: −0.79; 95% CI = −1.80, 0.22, P = 0.13); Fructosamine (WMD: −12.57; 95% CI = −40.74, 15.60; P = 0.38); Weight (WMD: −1.89; 95% CI = −4.55, 0.76; P = 0.16) and BMI (WMD: −0.12; 95% CI = −0.90, 0.65; P = 0.76).

The data showed significant reduction in FBG and improved insulin levels but limited effects were observed in other glycemic indexes. More extensive RCTs are required globally to achieve a holistic comprehension of the connection between berberis and T2DM.

## 1 Introduction

Glucose serves an essential role for the human body to maintain its functionality. Being the most prior option to fulfill the energy demands for vital reactions glucose needs to get in processing, for which insulin is integral [1]. Type 2 diabetes mellitus (T2DM) is a complex condition with multiple contributing factors, characterized by inadequate insulin production by pancreatic beta cells or resistance to insulin in the target organs [1]. As this deleterious condition interferes with the utility of multiple organs prominently causing end-stage renal disease, blindness, heart attacks and neuropathy, warranting a tight control of glycemic levels [2,3].

In recent years, there has been an alarming increase in the prevalence of diabetes cases. Researchers in India have predicted a massive rise in T2DM disease burden from 77 million to 134 million cases by 2045 [4]. Furthermore, according to WHO, in the year 2019 diabetes has engulfed two million lives globally [3], and is known to increase the risk of death by two-fold, posing a serious concern among practitioners to seek naive and upgraded solutions [5]. Treatment of T2DM evolved from lifestyle changes and diet to oral antidiabetics like metformin, sulfonylureas, thiazolidinediones and dipeptidyl peptidase-4 (DPP-4) inhibitors and eventually injectable glucagon-like peptide-1 (GLP-1) agonists and insulin taking over the treatment landscape [6].

Berberis vulgaris (BV) and berberis integerrima (BI), are plants found mainly in Europe and parts of South Asia with an active ingredient called berberine came to attention due to its anti-inflammatory, antimicrobial, antioxidant, and other beneficial cardiovascular attributes [[7], [8], [9], [10]]. At present, they are being explored for their favorable influence on glycemic indexes and insulin sensitivity making it a favorable choice for managing diabetes. Berberine is an ammonium salt found in berberis that decreases the intestinal absorption of glucose and inhibits carbohydrate hydrolyzing enzymes, thereby keeping glucose levels in check [11]. Their potential for managing T2DM can be evaluated by assessing their effects on fasting blood glucose (FPG), glycated hemoglobin (HbA1c), homeostatic model assessment for insulin resistance (HOMA-IR), and fasting serum insulin (FSI). Safari et al. supported BV being responsible for boosting insulin levels, meanwhile, no significant differences were reported in FBG, HbA1c %, and HOMA-IR [12]. Roshanravan et al. further endorsed BV being responsible for significantly reducing FBG against control [13]. Moreover, evidence has been found in the literature that supports BI's role in decreasing FBG, HOMA-IR, and HbA1c % in T2DM patients [10]. Sanjari et al. compared the efficacy of BI with metformin revealing similar effects yet no conclusive evidence on HOMA-IR was reported [9].

In addition, evidence highlights its cholesterol-lowering effects vouching for its use as an alternate or complement therapy [19]. The adverse effects of this herbal remedy are also minimal [12]. Additionally, BMI and weight are also closely knitted with T2DM, as Firouzi et al. showcases the positive impact of BV for reducing obesity or adipocytes, making it a double barreled gun [14]. An up-to-date clinical trial that depicted favorable consequences of BV on both lipid profile and glycemic indexes when compared with the placebo [15].

In this systematic review and meta-analysis, we will be evaluating the clinical efficacy and safety of BV and BI with regards to glycemic indexes, weight and body mass index (BMI) when compared with placebo. There has been a scarcity of meta-analyses addressing this aim and the already published literature have very small sample sizes with lack of recently published randomized controlled trials (RCTs) fading their significance [10]. In addition, Liang et al. conducted a study with all RCTs from China where the included studies showed high heterogeneity in terms of dosages, duration of treatment and combination of drugs [16]. In contrast, Kalmarzi et al. and Tabeshpour et al. evaluated only BV species while Sanjari et al. centered the focus towards exploring integerrima specie [[7], [8], [9]]. Current data shows a targeted approach oriented towards one single type of berberis creating a void to compare the species. Moreover, existing literature has an array of study designs, most of which are RCTs varying in their approaches and outcomes. Therefore, a comprehensive meta-analysis is necessary for the consolidation and assembly of disorganized data for fruitful outcomes. Our objective is to elucidate the potential benefits of berberis compared to existing treatment modalities, clarifying any ambiguities and providing a precise assessment of its clinical efficacy and safety as an adjunct therapy for T2DM. To the best of our knowledge, this will be the first meta-analysis to include two subtypes of berberis for managing T2DM, making it a valuable contribution to the field.

## 2 Materials and methodology

The Preferred Reporting Items for Systematic Reviews and Meta-Analysis (PRISMA) guidelines were meticulously followed while conducting this study [17]. The PRISMA checklist is reported in the supplementary material. Our Systematic review and Meta-analysis is registered on PROSPERO under the ID CRD42023463500.

## 3 Data sources and search strategy

Four electronic databases (PubMed, Google Scholar, ScienceDirect, and Cochrane CENTRAL) were searched for literature from their inception till September 24, 2023. The search string comprised of the following Medical Subject Headings (MESH terms): “berberis”, “barberry”, “berberis vulgaris”, “berberis integerrima”, “type 2 diabetes mellitus”, “insulin independent diabetes mellitus”, “T2DM”, “metabolic syndrome”, “metformin”. The complete search strategy used for each of the databases is provided in the Supplementary Table S1. The literature search was comprehensive with no time or location restrictions whatsoever. Gray and white literature search and records of unpublished literature search ensured that no study material was being overlooked.

## 4 Study selection and eligibility criteria

All the retrieved articles from the literature search were transferred to the Mendeley Desktop 1.19.8 (Mendeley Ltd., Amsterdam, Netherlands) to scan and eliminate the duplicates. The articles were further screened by two independent reviewers (HUH, AT) to match the PICO criteria. Study selection comprised of two phases. Initially, articles were chosen after reading their abstracts followed by full-read texts that helped finalize the studies. The following eligibility criterion was used: (a) T2DM patients; (b) T2DM patients receiving berberis (vulgaris/integerrima) therapy as intervention; (c) control group of placebo or metformin; (d) outcomes of interest such as FBG levels, HbA1c %, HOMA-IR, FSI, 2-h postprandial glucose (2hPPG), fructosamine, weight and BMI; (d) randomized controlled trials (RCTs). All the observational studies (cross-sectional, case-control, cohort), case reports, meta-analyses, reviews, and letters were excluded along with articles written in any language other than English. No dietary restrictions were placed as an inclusion criterion in our paper. Furthermore, articles that didn't strictly include vulgaris or integerrima subtypes of berberis were also scrapped from our paper.

## 5 Data extraction and quality assessment

Baseline characteristics data from each of the studies was extracted and recorded into a Microsoft Excel sheet. Characteristics included study design, region, sample size, duration of treatment, intervention, control, dosages, and outcomes assessed. Initially, two reviewers (HUH, EA) ensured the quality assessment of the included RCTs. This was followed by a third reviewer (AT) intervening to resolve any inconsistency in the risk of bias assessment independently. The Cochrane risk of bias tool 2.0 was used to assess the quality of RCTs based on the following five parameters: (i) Randomization process (ii) deviations from intended interventions (iii) missing outcome data (iv) measurement of outcome (v) selection of reported results. The risk of bias was categorized into low, high, or some concerns based on each of the above-mentioned domains [18].

## 6 Statistical analysis

All the baseline extracted data was uniformized to identical measuring units before performing the statistical analyses on Review Manager (Version 5.3. The Cochrane Collaboration). Inverse variance statistical method was applied and weighted mean difference (WMD) was the effect measure considered since all the outcomes assessed were continuous in nature. Additionally, a random-effects analysis model was used to pool the data and draw results while keeping the confidence interval (CI) value at 95%. Median values with their interquartile ranges (IQRs) were converted to means and standard deviations (SDs) for continuous data. Forest plots visualized the pooled results. Higgins I^2^ statistics analyzed the heterogeneity: a value of I^2^ = 25%–50% was considered statistically mild, 50%–75% as moderate, and >75% as severe heterogeneity. In plots with severe heterogeneity, sensitivity analysis was performed to lower the value of I^2^. A p-value of less than 0.05 was considered significant in all the cases.

## 7 Results

**Fig. 1:**
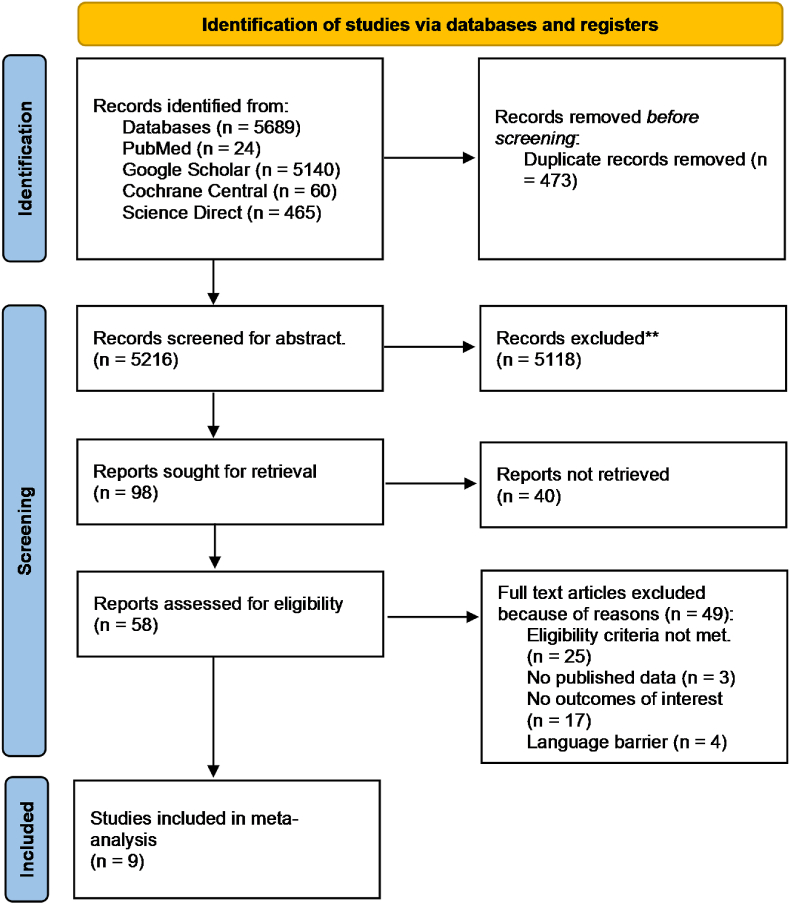
Prisma flow chart depicting details of literature search.

A total of 5689 articles were retrieved as the result of an initial literature search from 4 electronic databases. After removing duplicates, screening 5216 articles through the title and abstract excluded 5118 of them. 40 out of 98 articles left had to be eliminated as their reports were not retrieved. Full-text review of 58 studies allowed us to assess their eligibility criteria. Consequently, 9 RCTs were finalized as the other 49 had not met the eligibility criteria. A complete step-by-step literature search has been highlighted in the PRISMA flowchart in Fig. 1.

## 8 Study characteristics and patients’ baseline data

Study characteristics, patients’ baseline characteristics have been mentioned in detail in Table 1, Table 2 and Supplementary Table S2. A total of 547 patients were included in these 9 RCTs. Among them, 262 patients were part of the group that received berberis supplementation, 265 patients were in the control group, and 20 patients did not fall into any of our specified groups. In the intervention group, the mean age of the patients ranged from 47.6 years to 56.9 years with an average of 52.6 years. In the control group, the mean age of the patients ranged from 40.7 years to 57.6 years with an average of 50.4 years. The percentage of males ranged from 16.9% to 62% with a mean of 35.7% of total population. The percentage of females ranged from 38% to 83.1% with a mean of 53.2% of total population.

**Table 1:** Characteristics of the included studies

Author - Year	Study Design	Country	Subtype of Berberis	Duration of Treatment	Total Population - n	Dosage of Berberis	Outcome(s) Assessed	Risk of Bias
Shidfar et al., 2012 [19]	RCT	Iran	Vulgaris	12 weeks	42	3 gm daily	BMI, Weight, FSI, HbA1c, HOMA-IR	Moderate
Mansouri et al., 2017 [20]	RCT	Iran	NS	8 weeks	60	200 cc daily for 2 days followed by 100 cc for 8 weeks	HbA1c	Moderate
Lazavi et al., 2018 [21]	RCT	Iran	NS	8 weeks	46	200 ml of BJ daily	BMI, Weight, FBG, HbA1c	Low
Rashidi et al., 2018 [22]	RCT	Iran	Vulgaris	4 weeks	84	500 mg daily	BMI, FBG, HOMA-IR, Fructosamine	Low
Tahmasebi et al., 2019 [11]	RCT	Iran	Vulgaris	6 weeks	80	1000 mg dry extract & 157.3 mg berberine daily	FBG, Fructosamine	Low
Sanjari et al., 2020 [9]	RCT	Iran	Integerrima	12 weeks	80	480 mg daily	HbA1c, Weight, FBG, 2hPPG, Fructosamine	Low
Kermani et al., 2020 [23]	RCT	Iran	Vulgaris	3 weeks	50	550 mg daily	BMI, FBG	Low
Soltani et al., 2021 [10]	RCT	Iran	Integerrima	8 weeks	65	1000 mg daily	FBG, HbA1c, FSI, HOMA-IR, BMI	Moderate
Montazerifar et al., 2023 [15]	RCT	Iran	Vulgaris	8 weeks	40	1000 mg daily	BMI, Weight, FBG, HbA1c, FSI, HOMA-IR	Low

BMI, Body Mass Index; FSI, Fasting Serum Insulin; HbA1c, Glycated Hemoglobin; HOMA-IR, Homeostatic Model Assessment for Insulin Resistance; FBG, Fasting Blood Glucose; 2HPPG, 2 Hour-Postprandial Glucose; BJ, Barberry Juice; RCT, Randomized Controlled Trial; NS, Not Specified; mg, milligram; ml, milliliter.

**Table 2:** Population characteristics of the studies included

Author - Year	Gender - n (%)	No. of participants - Intervention	No. of participants - Control	Mean Age (SD) -Intervention	Mean Age (SD) -Control
Shidfar et al., 2012 [19]	NS	21	21	53.1 (6.3)	52.2 (4.9)
Mansouri et al., 2017 [20]	M = 37 (61.6%) F = 23 (38.4%)	30	30	48.2 (4.3)	48.2 (4.3)
Lazavi et al., 2018 [21]	M = 19 (41.3%) F = 27 (58.6%)	23	23	56.9 (8.5)	54 (6.6)
Rashidi et al., 2018 [22]	M = 34 (40.5%) F = 50 (59.5%)	42	42	50.2 (4.2)	45.1 (9.6)
Tahmasebi et al., 2019 [11]	M = 32 (40%) F = 48 (60%)	40	40	54.05 (8.0)	53.1 (7.7)
Sanjari et al., 2020 [9]	M = 25 (31.2%) F = 55 (68.8%)	30	30	51.8 (9.3)	46.5 (10)
Kermani et al., 2020 [23]	M = 31 (62%) F = 19 (38%)	26	24	47.6 (6.1)	40.7 (10.6)
Soltani et al., 2021 [10]	M = 11 (16.9%) F = 54 (83.1%)	30	35	56.1 (7.2)	57.6 (7.7)
Montazerifar et al., 2023 [15]	M = 11 (27.5%) F = 29 (72.5 %)	20	20	55.1 (8.1)	56.6 (10.2)

M, Male; F, Female; NS, Not Specified; SD, Standard Deviation.

## 9 Quality assessment

**Fig. 2:**
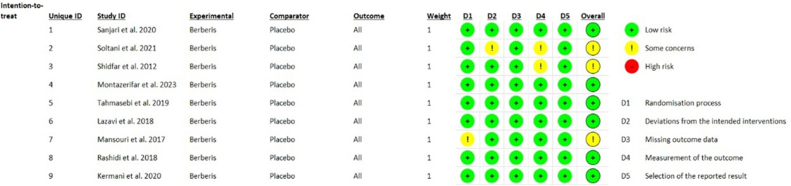
Risk of bias summary.

**Fig. 3:**
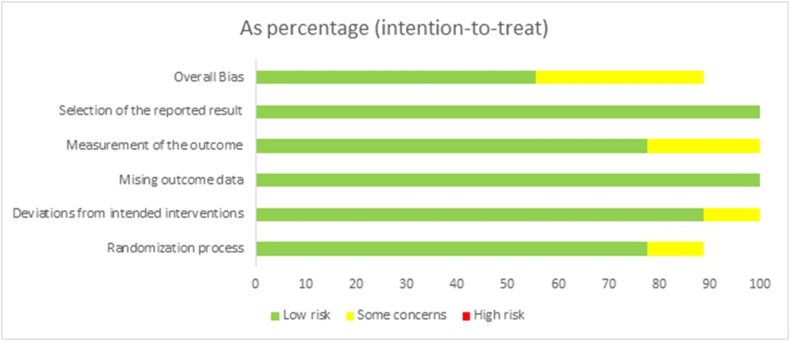
Graph depicting risk of bias.

According to the guidelines from Cochrane risk of bias tool 2.0, 6 trials reported low risk of bias and 3 trials reported moderate risk of bias (Fig. 2, Fig. 3). Trials with moderate risk of bias demonstrated some concerns in realms of randomization process, deviations from intended interventions and measurement of the outcomes. The PRISMA criteria were used to assess the meta-analysis’ quality.

## 10 Outcome analysis

We incorporated 9 trials (9–11,15,19–23), in our analysis which particularly collated changes in glycemic indexes and weight in diabetics following consumption of berberis. Our primary outcomes of interest included changes in FBG levels, HbA1c %, HOMA-IR, FSI levels, 2hPPG, and fructosamine levels from baseline. The secondary outcomes of interest consisted of changes in weight and BMI following berberis therapy.

### 10.1 Fasting Blood Glucose

**Fig. 4:**
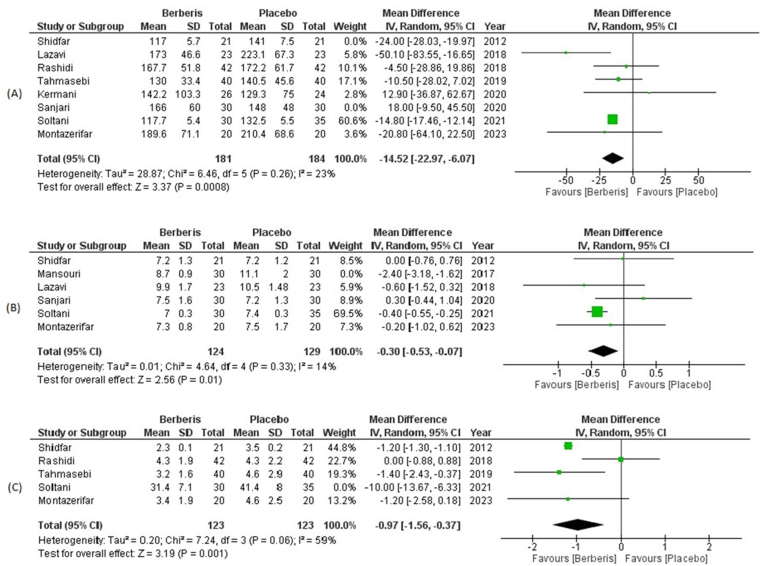
Post-sensitivity analysis forest plot depicting mean differences for (A) FBG levels (B) HBA1c % and (C) HOMA-IR in Berberis vs Placebo group.

FBG levels were evaluated by 8 including trials recruiting 232 diabetics in the berberis group and 235 diabetics in the placebo group. Upon administration of berberis extract, there was a statistically significant reduction in FBG in the intervention group (WMD: −15.16; 95% CI = −23.29, −7.03; P = 0.0003) when compared with patients under placebo (Supplementary Fig. S1). A sensitivity analysis was performed to gauge for potential outliers in the study which contributed to high heterogeneity. After excluding Shidfar et al. and Sanjari et al. the results were consistent in the berberis group (WMD: −14.52; 95% CI = −22.97, −6.07; P = 0.0008) and heterogeneity dropped from 74% to 23% (Fig. 4).

### 10.2 HbA1c %

Post-intervention HbA1c % was evaluated in 6 RCTs with 154 diabetic participants in the berberis group and 159 diabetic participants in the placebo group. HbA1c % reduced insignificantly upon administration of berberis extract vs. placebo group (WMD: −0.54; 95% CI = −1.15, 0.07; P = 0.09) (Supplementary Fig. S2). There was high heterogeneity observed among the included trials, therefore, we performed sensitivity to remove potential confounders in our study. Upon exclusion of Mansouri et al. the results showed a significant reduction in HbA1c % in the berberis group (WMD: −0.30; 95% CI = −0.53, −0.07; P = 0.01), and heterogeneity of our analysis declined from 83% to 14% (Fig. 4).

### 10.3 HOMA-IR

A total of 5 RCTs with 153 clinically diagnosed diabetics in the berberis group and 158 clinically diagnosed diabetics in the placebo group reported data for HOMA-IR levels from baseline. The pooled results established a significant reduction in HOMA-IR levels on consumption of berberis as compared to those without it (WMD: −1.57; 95% CI = −2.69, −0.45; P = 0.006) (Supplementary Fig. S3). A leave-one-out analysis was conducted to gauge for studies that contributed to high heterogeneity in results. Excluding Soltani et al. resulted in consistent findings (WMD: −0.97; 95% CI = −1.56, 0.37; P = 0.001) and a reduction in heterogeneity from 86% to 59% (Fig. 4).

**Fig. 5:**
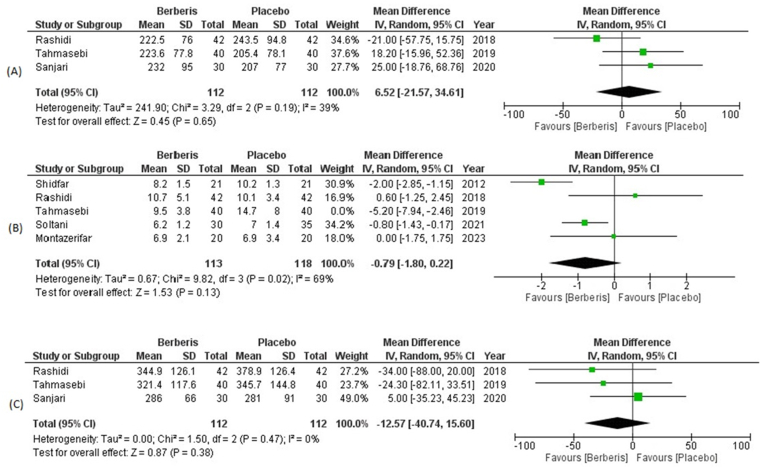
Forest plot depicting mean differences for (A) 2hPPG levels (B) FSI levels and (C) Fructosamine levels in Berberis vs Placebo group.

A total of 3 included trials assessed 2hPPG levels with a total of 224 participants (Berberis group: 112, Placebo group: 112). The pooled analysis revealed that consumption of berberis extract by diabetic patients had no significant reduction in 2hPPG levels from baseline when compared with the placebo group (WMD: 6.52; 95% CI = −21.57, 34.61; p = 0.65). A mild heterogeneity (I^2^ = 39%) was observed in pooled clinical data (Fig. 5).

### 10.4 FSI

FSI levels were evaluated from baseline by 5 trials enrolling 153 diabetics which were allocated to berberis extract and 158 diabetics allocated to placebo. The pooled analysis indicated a non-significant reduction in FSI levels in the intervention group as compared to the placebo group (WMD: −1.24; 95% CI = −2.46, −0.01; P = 0.05) (Supplementary Fig. S4). We performed sensitivity analysis due to high heterogeneity and upon exclusion of Tahmasebi et al. the results were consistently insignificant (WMD: −0.79; 95% CI = −1.80, 0.22, P = 0.13) with heterogeneity declining from 78% to 69% (Fig. 5).

### 10.5 Fructosamine

Fructosamine levels were assessed by 3 trials among a total of 112 clinically diagnosed diabetics in the berberis group and 112 clinically diagnosed diabetics in the placebo group. Consumption of berberis extract resulted in an insignificant reduction in fructosamine levels post-intervention (WMD: −12.57; 95% CI = −40.74, 15.60; P = 0.38) in comparison to placebo population. There was no heterogeneity (I^2^ = 0%) observed on pooled analysis of included trials (Fig. 5).

### 10.6 Weight

**Fig. 6:**
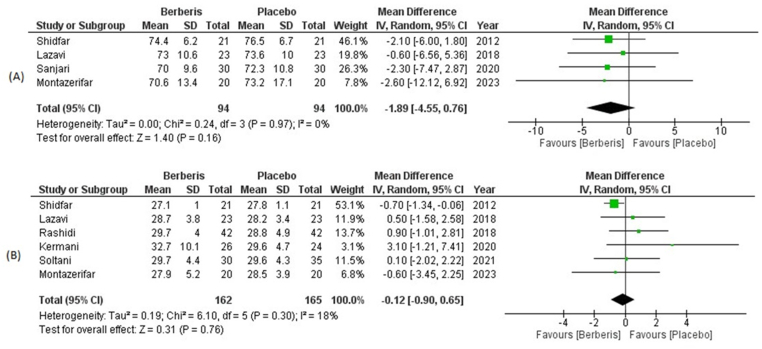
(A) Forest plot depicting mean differences for (A) weight and (B) BMI in Berberis vs Placebo group.

A total of 4 trials recruiting 94 clinically diagnosed diabetics in the berberis group and 94 participants in placebo assessed changes in weight post-intervention. Berberis extract consumption demonstrated a statistically insignificant decline in weight when compared to the placebo group (WMD: −1.89; 95% CI = −4.55, 0.76; P = 0.16). There was an absence of heterogeneity (I^2^ = 0%) in pooled analysis of included trials (Fig. 6).

### 10.7 Body Mass Index

A total of 6 clinical trials comprising 162 randomized diabetics in the berberis group and 165 randomized diabetics in the placebo group assessed reduction in BMI from baseline. The pooled analysis demonstrated no significant reduction in BMI following berberis consumption when compared with the placebo group (WMD: −0.12; 95% CI = −0.90, 0.65; P = 0.76). The analysis showed mild heterogeneity (I^2^ = 18%) upon pooling 6 trials which was best ignored (Fig. 6).

## 18 Discussion

In our present systematic review and meta-analysis, encompassing nine RCTs, we assessed the effectiveness of BV and BI in the management of T2DM patients. Regarding the impact of barberry consumption, our study findings revealed a substantial decrease in FBG, HbA1c, HOMA-IR levels. Moreover, the consumption of this extract had no discernible effects on 2hPPG, FSI, fructosamine, weight and BMI.

In our current study, a notable decrease in the FBG and HbA1c levels were observed following the administration of barberry. Previous research indicating the effectiveness of barberry consumption on glucose levels suggested that it may enhance glucose metabolism by promoting glycolysis, potentially as a result of inhibiting glucose oxidation in mitochondria [24]. Consequently, this process may lead to increased lactic acid production, activation of AMP-activated protein kinase (AMPK), and an increase in the adenosine monophospate/adenosine triphosphate (AMP/ATP) ratio. The activation of AMPK was suggested as the mechanism responsible for stimulating glucose uptake in muscle cells [24]. Furthermore, Pan et al. reported that the hypoglycemic effect of berberine may be related to the inhibition of sucrase-maltase activities and inhibition of α-glucosidase enzyme, thereby reducing glucose absorption from the intestine [25].

Our findings regarding the positive effect of berberis on HOMA-IR is consistent with Pierro et al.‘s study, as it suggests that berberine could effectively lower HOMA-IR levels by promoting increased insulin sensitivity [26], ultimately contributing to a decrease in insulin resistance, as reflected by the low HOMA-IR scores in our paper.

The results of our paper indicate that there was no statistically significant reduction in fructosamine levels. This null effect might be due to the fact, highlighted by Sanjari et al. [9], that fructosamine is a marker of short term blood glucose control, reflecting glucose levels over the past two to three weeks. In contrast, berberis primarily focuses on improving insulin sensitivity and reducing insulin resistance which may have a more substantial impact on long term markers such as HbA1c [27]. Safari et al. addressed a crucial ambiguity of whether the decrease in insulin levels are solely due to the therapeutic effects of berberis or just an aftermath of reduced FBG. Their results highlighted a substantial isolated reduction in insulin levels, without any appreciable changes in FBG levels [12], hence supporting the results of our paper.

In the field of herbal medicine or homeopathy, BV is also being used to dissolve calcium oxalate stones [28]. Jyothilakshmi et al. explored and reported the potency of berberis to reduce stone formation in kidneys. This attribute might be beneficial for diabetic patients suffering from renal stones [29], killing two birds with a single stone. Furthermore, this meta-analysis represents the first comprehensive examination of weight, BMI, 2hppg, fructosamine, and FSI as outcomes in the context of our current knowledge. Given the observed insignificant reduction upon the administration of berberis, we are unable to find any antecedent meta-analyses for comparative purposes.

## 19 Strengths and limitations

Our meta-analysis is the most up-to-date study, incorporating even the recently published RCTs from 2022 to 2023 which have been missed by other meta-analyses, to evaluate the effectiveness of berberis in managing T2DM.

The distinguishing point of our meta-analysis from previous papers is the inclusion of both berberis subtypes: Vulgaris and Integerrima. This approach allows for a more nuanced understanding of the effects of these subtypes on T2DM patients, enhancing the depth of our analysis. The outcomes are more likely attributed to the effects by BV since majority of the studies included this subtype. However, BI could have contributed significantly to the outcomes since its efficacy was evaluated by two studies. Berberis subtype in the remaining two studies remained unspecified by the authors. Our study did not evaluate the inter-specie differences in safety and efficacy of the subtypes of berberis. Hence, leaving room for further studies in future for evaluating which subtype is better in terms of long-term efficacy and safety.

In our efforts to produce robust results, we established stringent inclusion criteria and employed a meticulous search method. The sample size for each outcome in our meta-analysis is notably larger as compared to the already present literature. Therefore, our study provides a more comprehensive and reliable assessment of the impact of berberis on glycemic indices in patients of T2DM.

While our meta-analysis has a larger sample size compared to previous studies, it's important to acknowledge that it may still be considered relatively small for a meta-analysis, which might play a minor role in the value of significance of our findings. Another limitation to consider is that we were unable to conduct Egger's, Begg's, and Rank's correlation tests or generate funnel plots to assess publication bias due to the relatively small number of studies included in our analysis (9 RCTs), as these tests are typically performed with at least 10 or more studies. One of the limitations is that we had to exclude four RCTs from our meta-analysis due to the language barrier since they were available in Persian only.

Furthermore, it's important to note that every study included in our meta-analysis was exclusively conducted within Iran. This geographical limitation could potentially restrict the broader applicability of our findings and the relevance of our results. This highlights a dire need of more diverse, globally representative studies to provide a more comprehensive understanding of the topic.

## 20 Conclusion

In conclusion, our study suggests that while berberis therapy significantly improves insulin levels and reduces FBG levels, however, its effects on other glycemic indexes may be limited. Higher quality RCTs with longer follow-up periods are required to attain a comprehensive understanding of berberis' impact. To enhance the robustness and generalizability of future research, it's imperative to consider a more diverse and extensive range of studies from various regions of the globe, as opposed to confining research to a single country like Iran. This will further strengthen these findings and help achieve a more holistic comprehension of the connection between berberis and T2DM, potentially considering berberis as a novel therapeutic choice for the management of T2DM.

## Funding sources

The authors declare no funds were obtained for the completion of this research.

**Conflict of interest**

The authors declare that they have no known competing financial interests or personal relationships that could have appeared to influence the work reported in this paper.

## Data availability statement

No new datasets were generated to conduct this research. Publicly accessible data was obtained via systematic review to conduct this meta-analysis.

## Declaration of generative AI in scientific writing

No AI-assisted technologies were used in the writing process.

**Author contributions**

HUH: Conceptualization, Methodology, Analysis, Writing- Original draft preparation; EA: Data curation, Writing- Original draft preparation, Quality assessment; AT: Visualization, Conceptualization, Methodology, Writing- Original draft preparation; SMI: Writing- Original draft preparation, Data curation, Software; Reviewing and Editing MSA: Software, Validation, Reviewing and Editing; HF: Writing- Original draft preparation, Data curation.
